# Simultaneous transurethral resection of bladder cancer and prostate may reduce recurrence rates: A systematic review and meta-analysis

**DOI:** 10.3892/etm.2012.660

**Published:** 2012-08-09

**Authors:** SHENG LI, XIAN-TAO ZENG, XIAO-LAN RUAN, XING-HUAN WANG, YI GUO, ZHONG-HUA YANG

**Affiliations:** 1 Department of Urology, Zhongnan Hospital, Wuhan University, Wuhan, Hubei 430071;; 2 Department of Stomatology, Taihe Hospital, Hubei University of Medicine, Shiyan, Hubei 442000;; 3 Department and Institute of Hematology, Union Hospital, Tongji Medical College, Huazhong University of Science and Technology, Wuhan, Hubei 430022;; 4 The State Key Laboratory of Virology and Department of Epidemiology, School of Public Health, Wuhan University, Wuhan 430071, P.R. China

**Keywords:** simultaneous, benign prostate hyperplasia, bladder cancer, systematic review, meta-analysis

## Abstract

The aim of this study was to evaluate the recurrence rate of simultaneous transurethral resection of bladder cancer and prostate (TURBT+TURP) in the treatment of non-muscle invasive bladder cancer (NMIBC) with benign prostatic hyperplasia (BPH). We searched PubMed, the Cochrane Central Register of Controlled Trials (CENTRAL), EMBASE and the ISI Web of Knowledge databases from their establishment until March 2012, to collect all the original studies on TURBT+TURP vs. TURBT alone in the treatment of NMIBC with BPH. After screening the literature, methodological quality assessment and data extraction was conducted independently by two reviewers and meta-analysis was performed using the RevMan 5.1 software. The quality of data was assessed using the Grades of Recommendation, Assessment, Development and Evaluation (GRADE) approach. Eight studies, including seven non-randomized concurrent controlled trials (NRCCTs) and one randomized controlled trial (RCT), involving a total of 1,372 patients met the criteria. Meta-analyses of NRCCTs showed that in the TURBT+TURP group, overall recurrence rates were lower [odds ratio (OR), 0.76; 95% confidence interval (CI), 0.60–0.96; P=0.02] and the difference was statistically significant. The postoperative recurrence rate in the prostatic fossa/bladder neck (OR, 0.96; 95% CI, 0.64–1.45; P=0.86) and bladder tumor progression rates (OR, 0.96; 95% CI, 0.49–1.87; P=0.91) were similar between the TURBT+TURP and TURBT groups, but the difference was not significant. According to the GRADE approach, the level of evidence was moderate or low. Only one RCT demonstrated that overall postoperative tumor recurrence rates, recurrence rates at prostate fossa/bladder neck and bladder tumor progression rates between simultaneous groups and control groups were almost equal. There was no significant difference (P>0.05), and the level of evidence was moderate. For patients with NMIBC and BPH, simultaneous resection did not increase the overall recurrence rate of bladder tumors, it also did not cause metastasis and tumor progression, but it may reduce the recurrence rate. However, due to the low quality of investigations included in the present study, careful selection was necessary, and more large-scale and high-quality randomized controlled trials are also required for further confirmation.

## Introduction

Bladder cancer is the ninth most common malignancy worldwide; an estimated 386,300 new cases and 150,200 deaths from bladder cancer occurred in 2008 worldwide (1,2). The majority of bladder cancer occurred in males and among them, non-muscle invasive bladder cancer (NMIBC) accounted for 75–85% and the incidence rate was closely correlated to age (1,2). Benign prostate hyperplasia (BPH) is the most common cause of urination obstacles in elderly men; the incidence is also rising with the aging population (3). It is not unusual to encounter the clinical scenario of a male patient undergoing endoscopic treatment for bladder cancer (TURBT) who also requires transurethral resection of prostate (TURP). It was unclear whether it was safe to combine the two procedures since there was a risk of circulating cancer cells that may implant into the raw prostatic fossa and thereby enhance the risk of subsequent recurrences. In 1953 and 1956, simultaneous resection was first reported by Kiefer (4) and Hinman (5) based on four and three patients, respectively. The results indicated that simultaneous resection was inadvisable due to the high recurrence (100%) in the vesical neck or prostatic urethra. However, Greene and Yalowitz (6) in 1972 studied 100 patients who underwent simultaneous transurethral resection and the authors observed that simultaneous resection was preferable without increasing the risk of tumor recurrence. Since then, numerous studies on this issue have been conducted, however, the results of these studies were different or even contradictory (7). A previous meta-analysis (8), based on five pooled non-randomized concurrent controlled trials (NRCCTs) and one randomized controlled trial (RCT), reported a statistically significant result. NRCCT suffers more confounding factors and biases than RCT and they are not suitable for pooling, so the results were unconvincing.

It was unclear whether simultaneous resection of bladder tumor and prostate were safe and preferable for patients with NMIBC and BPH. An in depth reassessment of this issue may have important public health and clinical implications, so we performed this systematic review and meta-analysis to examine all the published evidence involving NRCCTs and RCTs, to provide unambiguous evidence whether simultaneous TURBT/TURP in the treatment of NMIBC with BPH was feasible.

## Materials and methods

### Literature search

A systematic search of the Cochrane Central Register of Controlled Trials (CENTRAL), PubMed, EMBASE and the ISI Web of Knowledge databases for the relevant published studies was conducted from their establishment to March 21, 2012. The relevant search terms were (‘prostatic hyperplasia’ OR ‘Benign Prostate Hyperplasia’ OR ‘prostate’) AND (‘simultaneous’ OR ‘simultaneously’ OR ‘synchronous’ OR ‘coinstantaneous’) AND (‘bladder tumor’ OR ‘bladder tumour’ OR ‘bladder cancer’ OR ‘bladder neoplasm’ OR ‘bladder carcinoma’ OR ‘vesical neoplasma’) AND (‘recurrence’ OR ‘relapse’). References were explored to identify relevant manuscripts. Only studies published in English were included.

### Study selection

A study was included in this systematic review when the following criteria were met: i) type of research: published RCT or NRCCTs; ii) participants: patients with NMIBC (including Ta, T1) combining benign prostatic hyperplasia (regardless of the severity, but excluding prostate cancer), and including information about patient age, length of follow-up and tumor stage; iii) interventions: simultaneous group (TURBT+TURP, resection of bladder tumor first, then prostate resection); control group (TURBT only), regardless of whether adjuvant chemotherapy was administered; iv) outcomes: overall tumor recurrence rates, recurrence rate at the prostatic urethra and/or bladder neck, and tumor progression and v) it was possible to obtain full texts.

### Methodological quality assessment

The methodological quality of each RCT was assessed using the Cochrane collaboration's tool for assessing risk of bias (9), which utilizes seven aspects: i) details of randomization method, ii) allocation concealment, iii) blinding of participants and personnel, iv) blinding of outcome assessment, v) incomplete outcome data, vi) selective outcome reporting and vii) other sources of bias, to provide a qualification of risk of bias.

For NRCCTs, we used MINORS (Methodological Index for Non-Randomized Studies) guidelines (10) to assess the methodological quality. MINORS guidelines consisted of 12 indexes: i) a clearly stated aim, ii) inclusion of consecutive patients, iii) prospective collection of data, iv) endpoints appropriate to the aim of the study, v) unbiased assessment of the study endpoint, vi) follow-up period appropriate to the aim of the study, vii) loss to follow-up less than 5%, viii) prospective calculation of the study size, ix) adequate control group, x) contemporary groups (control and studied group should be managed during the same time period, no historical comparison), xi) baseline equivalence of groups and xii) adequate statistical analyses, every item has two scores and the total score is 24; when the score is ≥16 points this indicates high quality, otherwise the quality is low (<16 points).

### Data extraction

Two researchers read the full texts independently and extracted the contents as follows: the sample inclusion criteria and sample size, methods and processes of sampling and grouping, basic information, interventions, outcome, length of follow-up, loss rates and reasons for the loss, and statistical methods of the studies. To obtain the missing information, authors were contacted by phone or e-mail. In studies involving RCT with multiple groups or non-randomized clinical trials, only the experimental and control groups associated with this study were extracted.

### Level of evidence

We evaluated the level of evidence by using the GRADE (Grades of Recommendation, Assessment, Development and Evaluation) approach (11). In addition, the GRADEprofiler 3.6 software (12) was used to create the evidence profile.

The GRADE system included: level of evidence: i) high quality (or A); further research is extremely unlikely to change our confidence in the estimate of effect, ii) moderate quality (or B); further research is likely to have an important impact on our confidence in the estimate of effect and may change the estimate, iii) low quality (or C); further research is extremely likely to have an important impact on our confidence in the estimate of effect and is likely to change the estimate and iv) very low quality (or D); we are extremely uncertain about the estimate.

### Statistical analysis

We proposed to pool results from single studies by meta-analysis where this was identified to be both clinically and statistically appropriate. We computed pooled ORs and 95% CIs using the Cochrane Review Manager 5.1 software (version 5.1.6) to generate forest plots and to assess the heterogeneity of the included studies. Heterogeneity was quantified by using the I^2^ statistic; low, moderate and high represented I^2^ values of 40, 70 and 100%, respectively. Where I^2^≤40% indicates there was no evidence of heterogeneity, the fixed-effects model was used, otherwise the random-effects model was used. In the presence of heterogeneity, we performed sensitivity analyses to explore possible explanations for heterogeneity and to examine the influence of various exclusion criteria on the overall risk estimate. We also investigated the influence of a single study on the overall risk estimate by removing each study in each turn, to test the robustness of the main results. Subgroup analysis was also conducted if significant heterogeneity was identified, according to methodological quality (low-quality studies vs. high-quality studies). Where possible, potential publication bias was assessed by visual inspection of the funnel plots of the primary outcome.

## Results

### Search results

**Figure 1:**
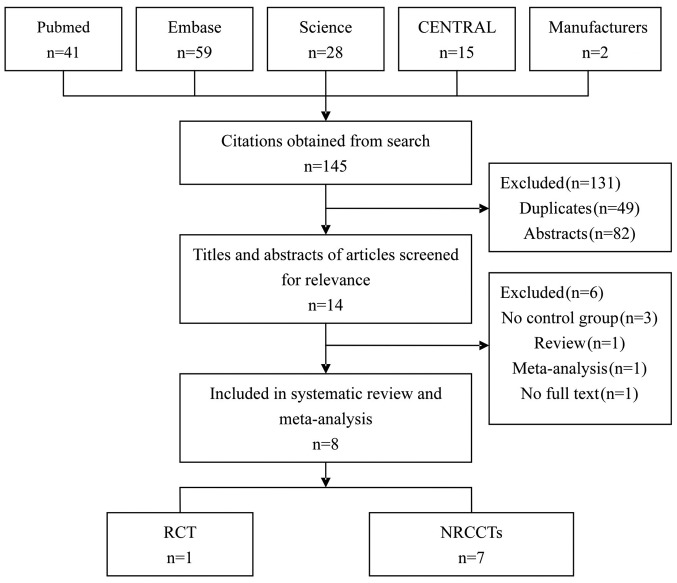
Flowchart for selecting RCTs and NRCCTs for the meta-analysis. RCT, randomized controlled trial; NRCCT, non-randomized concurrent controlled trial.

The initial search obtained 145 articles. After reading the abstracts and the full texts, 8 were selected for this study, including 1 RCT (13) and 7 NRCCTs (6,14–19). Fig. 1 shows the process of selection.

### Characteristics and quality of included studies

Table I shows the characteristics and quality points of each included study. Of the 8 studies, 3 were performed in the USA (6,14,15), 2 in Korea (17,18) and the remaining (13,16,19) in India, Turkey and Tunisia, respectively, during the period between 1972 and 2010, the total number of patients in each study ranged from 48 to 287. The baselines of 7 NRCCTs were similar and individual results are shown in Tables II and III. According to MINORS evaluation criteria (10), one study scored 22 points, 2 studies scored 20 points and 4 studies scored 19 points (Table I). The quality of RCTs according to the Cochrane Collaboration guidelines, provided a qualification of risk of bias. It refered to randomization only, lacking information with regard to allocation concealment and blind measurement; but no incomplete outcome data, no selective outcome reporting and other sources of bias, therefore there was a moderate risk of bias.

### Overall tumor recurrence rates

**Figure 2:**
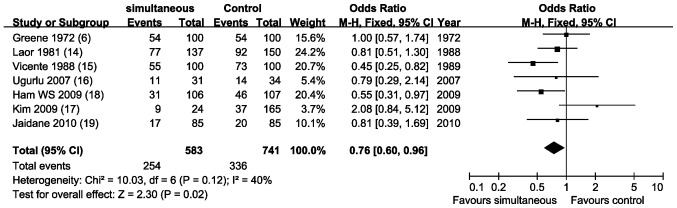
Overall tumour recurrence rates of pooled NRCCTs. NRCCT, non-randomized concurrent controlled trial; CI, confidence interval.

Meta-analysis of 7 NRCCTs (6,14–19) by a fixed-effects model (P=0.12; I^2^, 40%) revealed that simultanuous resection did not increase the recurrence rate of bladder tumor, on the contrary, recurrence rate was statistically lower than that in the control group (OR, 0.76; 95% CI, 0.60–0.96; P=0.02; Fig. 2).

The overall recurrence rate in simultanuous and control groups was 50 and 42.8%, respectively, (P>0.05) in the study by Singh *et al* (13).

### Recurrence rate at the prostatic urethra and/or bladder neck

**Figure 3:**
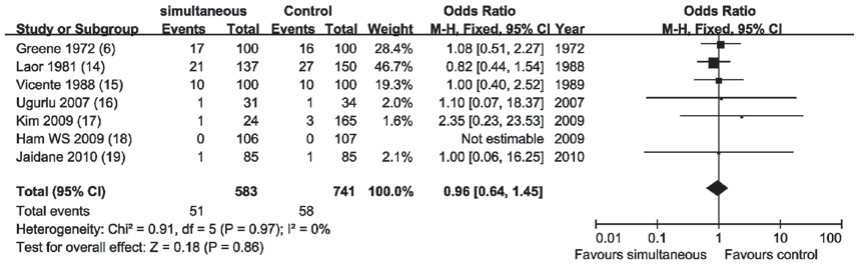
Recurrence rate at the prostatic urethra and/or bladder neck of pooled NRCCTs. NRCCT, non-randomized concurrent controlled trial; CI, confidence interval.

Meta-analysis of 7 NRCCTs (6,14–19) by a fixed-effects model (P=0.97; I^2^, 0%) showed that there was no statistical difference to compare recurrence rate to the prostatic urethra and/or bladder neck (OR, 0.96; 95% CI, 0.64–1.45; P=0.86; Fig. 3).

The recurrence rate at the prostatic urethra and/or bladder neck was 16.2 and 12.5%, respectively, (P>0.05) in the study by Sing *et al* (13).

### Tumor progression rates

**Figure 4:**
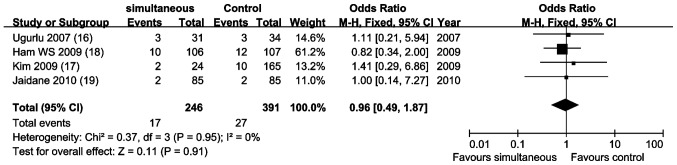
Tumor progression of pooled NRCCTs. NRCCT, non-randomized concurrent controlled trial; CI, confidence interval.

Meta-analysis of 4 NRCCTs (16–19) by a fixed-effects model (P=0.95; I^2^, 0%) showed that the tumor progression rates were similar and there was no statistical difference (OR, 0.96; 95% CI, 0.49–1.87; P=0.91; Fig. 4).

The tumor progression rate was 12.5 and 8.3%, respectively, (P=0.05) in the study by Singh *et al* (13).

### GRADE profile evidence

The included NRCCTs had the same three outcome indicators, they were the overall tumor recurrence rates, recurrence rate at bladder neck/prostatic fossa and tumor progression. The GRADE system evidence for each outcome level and reasons for upgrade and downgrade are shown in Table IV. Table IV also shows the GRADE quality of evidence for the included RCT.

## Discussion

Previous data have demonstrated that benign prostatic hyperplasia and other lower urinary tract obstructions were important factors in the pathogenesis of bladder cancer (20,21). In patients with benign prostatic hyperplasia, the retention of urine prolonged the duration of chemical carcinogens in bladder, and increasing the incidence of bladder cancer. Melicow *et al* (22) suggested that 4-aminobiphenyl and benzidine were decomposed into carcinogens, since the activity of urinary β-glucuronidase increased in patients with prostatic hyperplasia. This would cause bladder cancer. Due to the lower urinary tract obstruction, the bladder is susceptible to become infected, form stones and diverticulitis. Long-term and chronic irritation would cause the formation of epithelial hyperplasia and cystic or glandular cystitis. Part of the epithelium extended to the submucosal connective tissue formed von Brunn nests, this may become adenocarcinoma. As reported previously, these complications also stimulate transitional metaplasia and lead to squamous cell carcinoma. Therefore, early surgery over the same period to remove the lower urinary tract obstruction, not only does not increase the overall recurrence rate of bladder cancer, but also has the potential to reduce the recurrence rate (6,23).

In the past, patients with NMIBC and BPH were often treated with open or staging surgery. However, open surgery has certain shortcomings, including serious trauma, more postoperative complications and a longer time to recovery, particulary unbearable for elderly patients. Staging surgery would increase the risk of surgery and wasted money. With the development and popularity of urological endoscopic technology, numerous scholars now suggest simultanuous resection. However, in theory, simultanuous resection may increase the risk that cancer cells implant into the bladder neck and prostatic fossa. It was controversial whether simultanuous resection was feasible, although there were numerous associated studies.

There was a relevant meta-analysis published by Luo *et al* (8) in 2011, involving 6 studies and 983 patients. There was evidence that simultaneous TURBT/TURP did not increase the overall recurrence rate or recurrence rate in bladder neck/prostatic fossa. The shortcomings in this meta-analysis were as follows: i) incomplete retrieval or intended selective inclusion, so the efficiency of retrieval was low and would cause serious publication bias; ii) performed meta-analysis misused RR to pool the NRCCTs, which is the statistical index for prospective design (e.g. RCT); iii) failure to provide the risk bias figure, and failure to provide complete risk bias evaluation; iv) the methodological quality assessment tool for RCT was misused to assess the quality of NRCCTs, and failed to provide complete risk bias evaluation. In addition, the author also indicated in this paper that the size of inclusive and total samples were small. The results still require proof that includes larger sample size controlled clinical trials in the future, in order to obtain more accurate conclusions.

This study overcame the shortcomings of the previous study, based on a comprehensive literature search, evaluated RCT and NRCCTs using appropriate criteria, meta-analysis of NRCCTs, qualitative analysis of RCT, and the use of GRADE quality of evidence given in the standard classification. The results showed: simultaneous resection did not increase recurrence rate, on the contrary, the overall recurrence rate was lower than that of control group, it also did not increase the risk of tumor metastasis or tumor progression rate.

We also assessed the level of evidence using the GRADE approach. According to the GRADE approach, the quality of the evidence was only intermediate (the first two or three outcome indicators) and low (first outcome indicators) due to the limited evidence derived from combined NRCCT, and other reasons as follows: i) lack of allocation concealment and blinding and ii) the study controlled important confounding factors, but did not control others. RCTs were generally high quality, but this included one RCT with significant limitations of the study. Therefore, the quality of evidence was moderate in this RCT.

However, there were the following limitations in this meta-analysis. Firstly, we included only one RCT, so high quality meta-analysis of RCT could not be performed. Secondly, for non-randomized trials, the possibility of other bias reflected in the tumor status (single/multiple, tumor grade, associated with carcinoma *in situ*, etc.), postoperative bladder perfusion, technical surgical differences and transurethral tumor samples for inspection and other aspects of quality problems. Thirdly, the lack of long-term assessment of key indicators, such as the 5- or 10-year survival rate of patients. Lastly, the study sample size and overall sample size were small.

In summary, current evidence suggests that: for patients with NMIBC and BPH, simultanuous resection relief of the lower urinary tract obstruction, did not increase the overall recurrence rate of bladder tumors, but also did not cause metastasis and tumor progression, reduced expenses and shortened hospital stay and may reduce the relapse rate and improve the quality of life of patients. Based on the GRADE system, the quality of evidence, the recommended level was 2B. Due to the lack of evaluation of the system, further studies are required to be designed strictly according to CONSORT criteria (24), to design larger sample, high-quality, multi-center RCT, and include long-term key outcome indicators (such as 5- or 10-year survival rate of patients), in order to further evaluate the efficacy and safety of simultanuous resection.

## Figures and Tables

**Table I:** Characteristics of each primary study.

Study (ref.)	Year	Country of origin	Study type	Patients (n)		Mean age	Mean follow-up (months)	Outcome	Quality (points)
				Simultaneous group	Control group				
Greene and Yalowitz (6)	1972	USA	NRCCT	100	100	NA	132/132	1, 2, 4	High (19)
Laor *et al*(14)	1981	USA	NRCCT	137	150	71/60	69/96	1, 2, 4	High (19)
Vicente *et al*(15)	1988	USA	NRCCT	100	100	69/60	47/46	1, 2	High (20)
Ugurlu *et al*(16)	2007	Turkey	NRCCT	31	34	55.97/68.22	30.6/27.4	1, 2, 3	High (19)
Kim *et al*(17)	2009	Korea	NRCCT	24	165	70/64.1	52.2/43.8	1, 2, 3	High (19)
Ham *et al*(18)	2009	Korea	NRCCT	106	107	66.7/65.5	50.1/54.3	1, 2, 3, 4, 5	High (22)
Jaidane *et al*(19)	2010	Tunisia	NRCCT	85	85	71/71	35.2/33.1	1, 2, 3	High (20)
Singh *et al*(13)	2009	India	RCT	24	24	56.06/57.36	35.7/37.6	1, 2, 3	Moderate

1, Overall tumor recurrence rates;

2, recurrence rate at the prostatic urethra and/or bladder neck (metastasis is considered to be planted);

3, tumor progression;

4, single or multiple tumors were relative to recurrence rate;

5, Qmax and PVR volume (postvoid residual urine volume) at the first 3-months; RCT, randomized controlled trial; NRCCT, non randomized concurrent control trial; NA, not available.

**Table II:** Characteristics of the simultaneous groups.

Study	Year	Patients (n)	Total recurrence n (%)	Recurrence in bladder neck and/or prostatic fossa, n (%)	Progression (%)	Single/multiple (n)	Ta/T1 (n)	Grade (n)	Adjuvant chemotherapy
Greene and Yalowitz (6)	1972	100	54 (54)	17 (17)	NA	81/19	NA	57/29/14^a^	NA
Laor *et al*(14)	1981	137	77 (56.2)	21 (15)	NA	112/25	NA	34/35/51^a^	NA
Vicente *et al* (15)	1988	100	55 (55)	10 (10)	NA	58/42	21/79	4/78/18^a^	NA
Ugurlu *et al* (16)	2007	31	11 (35.5)	1 (3.2)	3 (9.7)	31/0	25/6	26/3/2^a^	N
Kim *et al* (17)	2009	24	9 (37.5)	1 (4.2)	2 (8.3)	NA	8/16	13/11^b^	NA
Ham *et al* (18)	2009	106	31 (29.2)	0	10 (9.4)	58/48	21/85	60/46^b^	Y
Jaidane *et al* (19)	2010	85	17 (20)	1 (1.2)	2 (2.3)	70/15	9/76	32/45/8^a^	Y
Singh *et al* (13)	2009	24	12 (50)	4 (16.2)	3 (12.5)	24/0	17/7	10/11/3^a^	N

a WHO1973 pathological grading system of non-muscle invasive urothelial neoplasms: grade1/2/3;

b WHO2004 pathological grading system of non-muscle invasive urothelial neoplasms: gradelow/high; Y, yes; N, no; NA, not available.

**Table III:** Characteristics of the control groups.

Study	Year	Patients (n)	Total recurrence n (%)	Recurrence in bladder neck and/or prostatic fossa, n (%)	Progression (%)	Single/multiple (n)	Ta/T1(n)	Grade (n)	Adjuvant chemotherapy
Greene and Yalowitz (6)	1972	100	54 (54)	16 (16)	NA	77/23	NA	59/23/18^a^	NA
Laor *et al*(14)	1981	150	92 (61.3)	27 (18)	NA	124/26	NA	35/7/57^a^	NA
Vicente *et al*(15)	1988	100	73 (73)	10 (10)	NA	52/48	24/76	18/73/9^a^	NA
Ugurlu *et al*(16)	2007	34	14 (41.2)	1 (2.9)	3 (8.8)	34/0	25/9	31/3/0^a^	N
Kim *et al*(17)	2009	165	37 (22.4)	3 (1.8)	10 (6.1)	NA	43/109	81/84^b^	NA
Ham *et al*(18)	2009	107	46 (43.0)	0	12 (11.2)	56/51	19/88	59/48^b^	Y
Jaidane *et al*(19)	2010	85	20 (23.5)	1 (1.2)	2 (2.3)	65/20	11/74	33/44/8^a^	Y
Singh *et al* (13)	2009	24	11 (42.8)	3 (12.5)	2 (8.3)	24/0	18/6	9/11/4^a^	N

a WHO1973 pathological grading system of non-muscle invasive urothelial neoplasms: grade 1/2/3;

b WHO2004 pathological grading system of non-muscle invasive urothelial neoplasms: grade low/high; Y, yes; N, no; NA, not available.

**Table IV:** GRADE profile evidence of the included studies.

Quality assessment							No. of patients		Effect			
No. of studies	Design	Risk of bias	Inconsistency	Indirectness	Imprecision	Other considerations	Simultaneous	Control	Relative (95% CI)	Absolute	Quality	Importance
Recurrence												
7	NRCCT	Serious^a^	No serious inconsistency	No serious indirectness	No serious imprecision	Strong association^b^	254/583 (43.6%)	336/741 (45.3%)	OR 0.76 (0.6–0.96)	67 fewer/1000 (from 10 fewer to 121 fewer)	⊕⊕○○ Low	Critical
1	RCT	Serious^a^	No serious inconsistency	No serious indirectness	No serious imprecision	None	12/24 (50%)	11/24 (45.8%)	RR 1.09 (0.6–1.97)	41 more/1000 (from 183 fewer to 445 more)	⊕⊕⊕○ Moderate	Critical
Recurrence rate at the prostatic urethra and/or bladder neck												
7	NRCCT	Serious^a^	No serious inconsistency	No serious indirectness	No serious imprecision	Strong association^b^, increased effect for RR ∼1^c^	51/583 (8.7%)	58/741 (7.8%)	OR 0.96 (0.64–1.45)	3 fewer/1000 (from 27 fewer to 31 more)	⊕⊕⊕○ Moderate	Critical
1	RCT	Serious^a^	No serious inconsistency	No serious indirectness	No serious imprecision	None	4/24 (16.7%)	3/24 (12.5%)	RR 1.33 (0.33–5.33)	41 more/1000 (from 84 fewer to 541 more)	⊕⊕⊕○ Moderate	Critical
Progression												
4	NRCCT	Serious^a^	No serious inconsistency	No serious indirectness	No serious imprecision	Strong association^b^ increased effect for RR ∼1^c^	17/246 (6.9%)	27/391 (6.9%)	OR 0.96 (0.49–1.87)	3 fewer/1000 (from 34 fewer to 53 more)	⊕⊕⊕○ Moderate	Important
1	RCT	Serious^a^	No serious inconsistency	No serious indirectness	No serious imprecision	None	3/24 (12.5%)	2/24 (8.3%)	RR 1.5 (0.27–8.19)	42 more/1000 (from 61 fewer to 599 more)	⊕⊕⊕○ Moderate	Important

a Evidence limited by study design and implementation; lack of allocation concealment and blinding;

b the study controlled important confounding factors, studies have shown that the effect is significant and the results are consistent;

c OR=0.96. RCT, randomized controlled trial; NRCCT, non-randomized concurrent controlled trial; CI, confidence interval; OR, odds ratio; RR, relative risk; GRADE, Grades of Recommendation, Assessment, Development and Evaluation.
